# Nonpharmacological interventions to improve the sleep of informal caregivers of people with dementia: a systematic review and meta-analysis

**DOI:** 10.1093/geront/gnag081

**Published:** 2026-04-29

**Authors:** Shanshan Wang, Yuqian Luo, Jiaming Xiong, Yuting Xia, Sze Him Isaac Leung, Patricia M Davidson

**Affiliations:** School of Nursing, Faculty of Health and Social Sciences, The Hong Kong Polytechnic University, Hong Kong SAR, China; School of Nursing, Faculty of Health and Social Sciences, The Hong Kong Polytechnic University, Hong Kong SAR, China; School of Nursing, Faculty of Health and Social Sciences, The Hong Kong Polytechnic University, Hong Kong SAR, China; School of Nursing, Faculty of Health and Social Sciences, The Hong Kong Polytechnic University, Hong Kong SAR, China; Department of Statistics, The Chinese University of Hong Kong, Hong Kong SAR, China; Faculty of Medicine and Health, International Centre for Future Health Systems, University of New South Wales, Randwick, Australia

**Keywords:** Caregivers, Dementia, Sleep, Systematic review, Nursing

## Abstract

**Background and Objectives:**

Sleep problems are common among informal caregivers of people with dementia. Although various nonpharmacological interventions developed to improve caregivers’ sleep, comprehensive syntheses of their categories and effectiveness are lacking. This study synthesizes the types of nonpharmacological interventions used for this population and systematically evaluates their effects on sleep outcomes.

**Research Design and Methods:**

Six databases (Medline, Scopus, Web of Science, Embase, PsycINFO, and CINAHL) were systematically searched for studies evaluating nonpharmacological interventions and reporting sleep outcomes in informal dementia caregivers. Two researchers independently selected studies and assessed quality.

**Results:**

Of 4,147 records identified, 30 studies were synthesized and 22 meta-analyzed. Seven intervention categories were identified: multicomponent behavioral sleep interventions, sensory-based interventions, physical exercise, institutional respite care, cranial electrical stimulation, caregiver education, and nighttime monitoring systems. Pooled analyses indicated nonpharmacological interventions significantly improved overall sleep quality (SMD = −0.60, 95% CI = −1.12 to −0.08) and insomnia (SMD = −0.43, 95% CI = −0.83 to –0.04). Multicomponent behavioral interventions specifically improved overall sleep quality (SMD = −0.36, 95% CI = −0.61 to −0.12). Research on other specific sleep parameters remains limited.

**Discussion and Implications:**

Nonpharmacological interventions may effectively improve overall sleep quality and insomnia among informal caregivers of people with dementia. However, significant research gaps persist regarding the effectiveness of these interventions in improving specific sleep parameters. Since heterogeneity among the included studies remains high, caution is warranted when interpreting the results in practice. Future large-scale and methodologically rigorous trials are needed.

## Background

With the escalating global macroeconomic burden of dementia, projected to cost the world economy 14,513 billion international dollars from 2020 to 2050 (Chen et al., 2024), informal caregivers of people with dementia have become vital health providers in communities worldwide. As of 2018, these caregivers provided over 18.5 billion hours of informal care, a figure that has likely increased due to global population aging (Alzheimer’s Association, 2019). Because of the intensive caregiving tasks and progressive decline in various functional abilities in people with dementia, caregivers experience significant psychological, physical, and financial burdens (Tahami Monfared et al., 2022), which results in most informal caregivers suffering from physical and mental health challenges, including sleep problems (Nemcikova et al., 2023; Osakwe et al., 2022). Among these challenges, sleep problems are the most prevalent, with data indicating that 50%–70% informal caregivers face sleep issues, making it a critical health concern for this population (Huisman et al., 2025). A systematic review has shown that, compared to age-matched non-caregiver adults, caregivers of people with dementia experience shorter sleep durations, equivalent to losing 2.42 to 3.5 hours per week, and their sleep quality is significantly poorer (Gao et al., 2019). Additionally, impaired sleep parameters, such as prolonged sleep onset latency, extended wake after sleep onset, and frequent nocturnal awakenings, are also commonly reported among this population (Brewster et al., 2024).

Poor sleep has significant negative effects on the health of dementia caregivers. While short-term sleep disturbance can contribute to or exacerbate symptoms of anxiety, depression, and fatigue, chronic sleep problems are associated with an increased risk of stroke, obesity, diabetes, hypertension, and cardiovascular disease in informal caregivers (Cooper et al., 2022; Wang et al., 2024). Besides, sleep problems can impair caregivers’ cognitive performance, which may potentially compromise the quality of care they provide and negatively affect the quality of life for people with dementia (Cooper et al., 2022). Hence, implementing effective interventions to improve sleep for informal caregivers of people with dementia is essential.

Due to the side effects associated with pharmacological interventions, nonpharmacological approaches are increasingly utilized to address sleep problems (Javed et al., 2023). Specifically, various types of nonpharmacological interventions have been investigated to improve the sleep of informal caregivers of people with dementia, including behavioral sleep interventions that modify sleep-related behaviors, sometimes using principles from cognitive behavioral therapy for insomnia (Song et al., 2024), caregiver education on caregiving skills (Park et al., 2020), mindfulness meditation (Molero Jurado et al., 2020), etc. However, significant variabilities exist in the nature of these interventions, and there is currently a lack of comprehensive synthesis regarding the types and detailed components of nonpharmacological interventions used for this population. This gap hinders the ability to provide informed recommendations for clinical practice. Furthermore, while a previous review indicates potential benefits of such interventions for caregivers’ sleep (Gao et al., 2019), a critical synthesis of their effects remains insufficient. Additionally, sleep health is a multidimensional construct that can include overall sleep quality and specific sleep parameters such as total sleep time, sleep efficiency, sleep latency, wake after sleep onset, and sleep architecture (Buysse, 2014). These constructs can be measured using self-reported questionnaires or sleep diaries, as well as objective measures such as actigraphy or polysomnography. Given that different interventions may differentially affect specific aspects of sleep and that subjective and objective measures do not always converge (Li et al., 2025). The absence of clear evidence regarding the effects of nonpharmacological interventions on these specific sleep outcomes limits the strength of evidence-based recommendations. Therefore, a systematic review and meta-analysis, incorporating proper subgroup analyses, is essential to provide robust guidance for both research and practice.

### Objectives

The objectives of this study were to (1) synthesize the various types of nonpharmacological interventions applied in informal caregivers of people with dementia to improve their sleep, and (2) determine the effects of nonpharmacological interventions on caregivers’ sleep outcomes across a range of sleep indices (e.g., overall sleep quality, sleep latency, wake after sleep onset, sleep duration, and insomnia symptoms).

### Research questions

1. What types of nonpharmacological interventions have been used to improve sleep among informal caregivers of people with dementia?
2. What are the effects of nonpharmacological interventions on caregivers’ sleep outcomes across a range of sleep indices (e.g., overall sleep quality, sleep latency, wake after sleep onset, sleep duration, insomnia symptoms)?

## Methods

### Study design

This study is a systematic review and meta-analysis. The protocol has been registered with PROSPERO (ref: CRD42024594090). The review was reported in accordance with the Preferred Reporting Items for Systematic Reviews and Meta-Analyses (PRISMA) extension statement (Moher et al., 2010).

### Literature search strategies

Six databases were systematically searched on December 9, 2024, including MEDLINE, Embase, PsycINFO, Scopus, CINAHL, and Web of Science. An updated search was conducted on January 9, 2026. Keywords included dementia (dement* OR Alzheimer* OR “cognitive dysfunction” OR “neurocognitive disorders” OR “Lewy Body Disease”), informal caregiver (carer* OR caregiv* OR care-giv* OR famil* OR spouse* OR relative* OR unpaid), nonpharmacological intervention (nonpharmacological intervention* OR cognitive training OR behavior therapy OR muscle relaxation OR autogenic training OR phototherapy OR mindful* OR psychoeducat* OR music* OR training OR exercise OR cognitive behavi* OR psychotherapy OR counseling OR case management OR family therapy), sleep problems (sleep*OR insomnia* OR sleepless* OR “dyssomnia” OR “early awakening” or parasomnia). A manual search was performed by reviewing the reference lists of included studies.

### Selection criteria

The inclusion criteria of studies were based on the Population, Intervention, Comparison, Outcome, and Study design (PICOS) framework of the research questions: studies that included (1) P: Informal caregivers who are unpaid and responsible for caring for the daily life of a loved one with any types of dementia at any stage (e.g., mild, moderate, severe); (2) I: any type of nonpharmacological interventions to improve sleep in dementia caregivers; (3) C: with or without a control group; (4) O: any sleep-related measures, such as overall sleep quality, sleep efficiency (percentage of time spent lying in bed at night that is actually asleep), sleep latency (the amount of time it takes to fall asleep after going to bed), total nighttime sleep duration, wake after sleep onset, and insomnia; (5) S: randomized controlled trials (RCTs) or quasi-experimental studies that were conducted at community or home settings.

Exclusion criteria included: (1) studies mixing informal caregivers with other populations without separately reporting the findings; (2) studies implementing an intervention to the patient, without any intervention to the caregiver; (3) publications without sufficient data, such as conference proceedings and trial registries.

### Data screening and extraction

Covidence, a web-based platform, was used for data screening. Each search record was independently screened by two researchers against the inclusion and exclusion criteria (Authors S. W., Y. L., J. X., and Y. X.). To improve accuracy and reproducibility, all raters were provided with a detailed screening manual with definitions and examples of eligible criteria, minimizing subjective interpretation. The inter-rater reliability for the screening process could be automatically calculated by Covidence. Full-text screening was then conducted by the same researchers independently. If any discrepancies arose, a third researcher (S. W.) was consulted to reach consensus. Data were extracted independently from the included studies according to the same principle, including author names, publication year, country, study design, sample size, average age, intervention and control group details, sleep outcome variables, statistical values, measurement tools, and main findings of each study.

### Quality appraisal

The Cochrane Risk of Bias tool for randomized trials (RoB 2) (Higgins et al., 2011) was used to evaluate the quality of randomized controlled trials, and the Risk of Bias in Nonrandomized Studies of Interventions tool (ROBINS-I v2) was used to assess the quality of quasi-experimental studies. RoB 2 assesses five domains: randomization process, deviations from intended interventions, missing outcome data, outcome measurement, and selection of reported results. Meanwhile, ROBINS-I v2 evaluates seven domains: bias due to confounding, participants’ selection, interventions’ classification, deviations from intended interventions, missing data, measurement of outcomes, and selection of reported results. Based on the answers to signaling questions in each domain, RoB 2 assesses the risk of bias as low, some concerns, or high risk, while ROBINS-I v2 assesses it as low, moderate, serious, or critical. Each paper was rated by two researchers (Y. L., J. X., and Y. X.) independently, and discrepancies were resolved by discussing with a third researcher (S. W.).

### Data analysis

Qualitative synthesis was used to categorize the types of nonpharmacological interventions and the main findings of each study. Quantitative data, such as standardized mean difference (SMD) and 95% confidence intervals (95% CI), were calculated to measure the pooled effect in the meta-analyses. To calculate between-group effect sizes for the meta-analysis, we extracted post-intervention data for both the intervention and control groups. Specifically, we extracted the post-treatment mean, standard deviation, and sample size for each group. The intervention group was defined as the arm receiving sleep therapy, while the “control group” was defined as the arm receiving either an active control (e.g., treatment-as-usual) or a passive control (e.g., waitlist, no treatment). For studies lacking a control group (i.e., single-arm pre-post designs), we extracted baseline and post-intervention data and treated the baseline data as the control. Specifically, the means, standard deviations, and sample sizes at baseline and post were extracted. Given the diverse intervention components, durations, and measurement methods utilized across the studies analyzed, we used random-effects models (restricted maximum likelihood random effects) to estimate pooled effects (Langan, 2022). This model accounted for heterogeneity stemming from differences in study design, implying distinct true effect sizes among studies, in contrast to a fixed effects model (Borenstein et al., 2009). For the calculation of different sleep indices, scores from each study were recorded in the same direction so that lower scores indicated higher sleep quality. According to Cohen (Cohen, 1988), SMD cutoff points of 0.20, 0.50, and 0.80 are considered to represent a small, moderate, and large effect, respectively. Subgroup analysis for different intervention types or measure types (i.e., subjective and objective measures) and sensitivity analyses by excluding nonrandomized studies were conducted where applicable. Hedges’ *g* was used to interpret the effect sizes as follows: 0.15 indicates a small effect, 0.40 a medium effect, and 0.75 a large effect (Brydges, 2019). The analysis was conducted using R software. All *p*-values were two-tailed, with values below .05 regarded as statistically significant.

## Results

**Figure 1:**
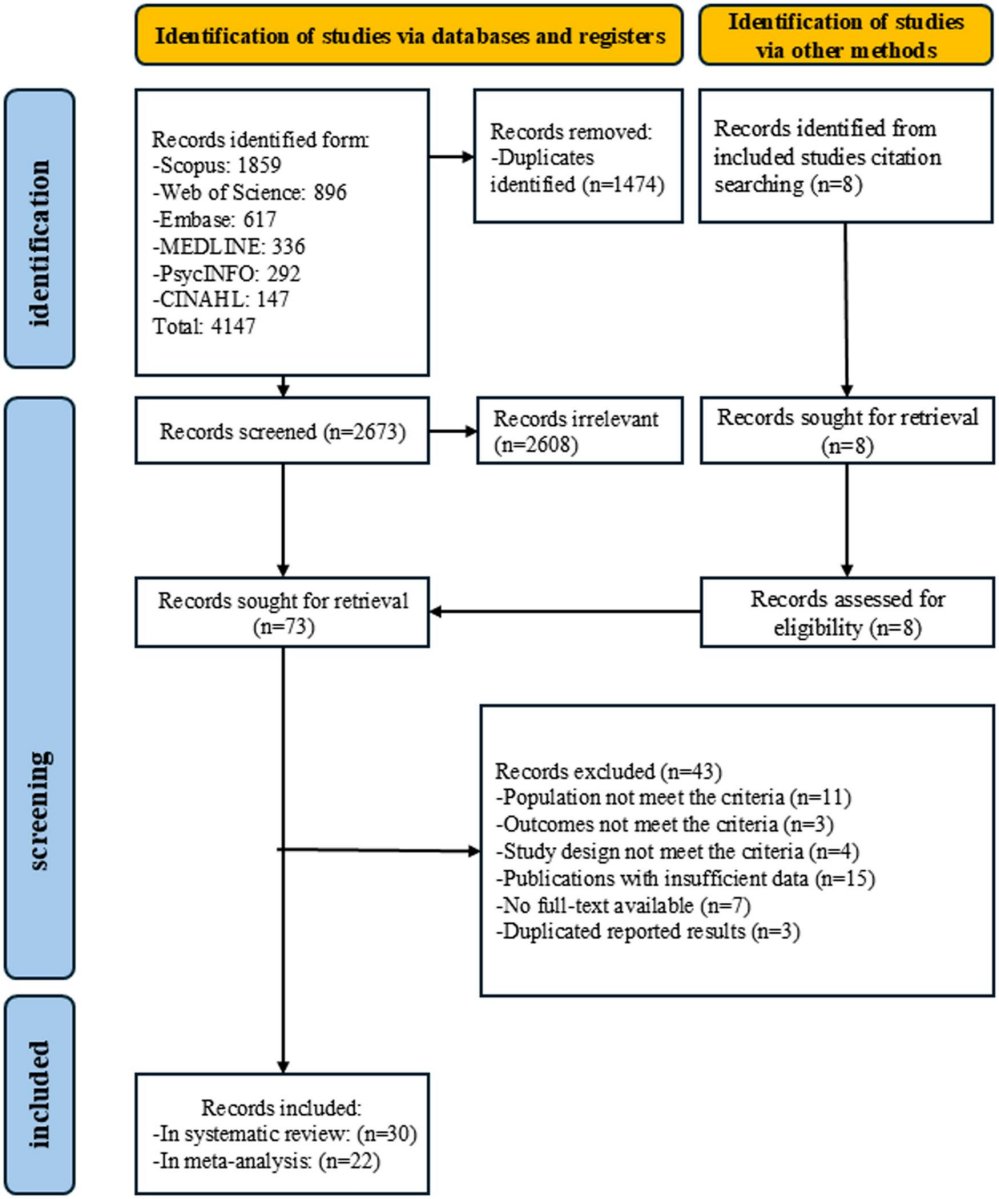
The PRISMA flow diagram.

A total of 4,147 articles were retrieved from the database search, with 30 included in this systematic review after screening and 22 included in the meta-analysis. The Cohen’s Kappa (*k*) of inter-rater reliability during the full-text screening process was from 0.74 to 0.79, indicating substantial agreement (Landis & Koch, 1977). Details can be found in Figure 1.

### Characteristics of included studies

The included studies (*n* = 30), primarily from the United States of America (*n* = 21), involved 1,988 participants, with 19 RCTs and 11 quasi-experimental designs. The sleep outcomes measured included overall sleep quality [Pittsburgh Sleep Quality Index (PSQI), *n* = 16], sleep efficiency (*n* = 11), sleep latency (*n* = 8), sleep duration (*n* = 7), wake after sleep onset (*n* = 4), and insomnia (*n* = 5). Actigraphy was used to measure sleep in 10 studies. Among 30 studies, the baseline data in nine single-arm pre-post designs and one crossover design were considered as the control group. For other studies with a control group, there are 10 passive controls (including six usual care, two waitlist, and two no treatment) and 10 active controls (including four educational sessions or materials, two attention controls, one respite session, one cognitive appraisal training, one sham CES, and one partially activated in-home technology). Eight studies were not included in the meta-analysis due to limited data availability and the inability to access the original data (Akkerman & Ostwald, 2004; Fowler et al., 2016; Livingston et al., 2019; Park et al., 2020; Rowe et al., 2010; Scheffer et al., 2025; Shih et al., 2024; Tewary et al., 2018). See Table 1.

**Table 1:** Characteristics of the included studies.

Author (years)	Country	Study design	Caregivers’ age (sample size)		Intervention duration	Intervention		Sleep outcome measures	Main findings/results
			IG	CG		Experimental	Control		
Akkerman et al. (2004)	United States	RCT	18	17	10 weeks	Cognitive behavioral therapy	Waitlist	Total minutes scored as awake, number of wake episodes, longest wake episodes (A)	Fewer total minutes awake, fewer wake episodes, and less time spent during each wake episode
Ali et al. (2015)	Pakistan	Within-subjects design	52.38 ± 15.54 (*n* = 8)		5–8 weeks involving 10 sessions	Cognitive behavioral therapy including sleep hygiene components	–	Insomnia (a subscale in General Health Questionnaire-28)	Significant improvements on insomnia
Brewster et al. (2023)	United States	RCT	IG1: 66.1 ± 10.4 (*n* = 51) IG2: 63.5 ± 11.3 (*n* = 59)	65.00 ± 8.3 (*n* = 27)	7 weeks	Caregiving and self-care training	Usual care	Overall sleep quality (PSQI); insomnia (ISI)	Overall sleep quality and insomnia improved, but nonsignificant time-by-group interaction effects (3 and 6 months)
Elliott et al. (2010)	United States	RCT	62.3 ± 12.1 (*n* = 257)	60.2 ± 12.9 (*n* = 238)	24 weeks	Caregiving and self-care training	Educational materials support	Overall sleep quality (self-report)	Overall sleep quality improved, but the time-by-intervention interaction effect was not statistically significant
Figueiro et al. (2015)	United States	Quasi-experimental study	71.8 ± 12.3 (*n* = 34)		4 weeks	Light therapy	–	Sleep duration (A); sleep score (A); overall sleep quality (PSQI)	Overall sleep quality improved, but the changes did not reach statistical significance
Fowler et al. (2016)	United States	RCT	60 (*n* = 28)		16 weeks	An interprofessional virtual healthcare neighborhood	Usual care	Insomnia (ISI); sleep interruptions (A)	Failed to improve insomnia and sleep interruptions
Hirano et al. (2011)	Japan	RCT	72.6 ± 4.0 (*n* = 17)	75.0 ± 4.6 (*n* = 14)	12 weeks	Physical exercise	Nonexercise	Overall sleep quality (self-report)	Significantly improved the overall sleep quality at 12 weeks but failed to improve depression
Jain et al. (2014)	United States	Quasi-experimental study	64 ± 7 (*n* = 10)		8 weeks	Mindfulness meditation	–	Insomnia (ISI)	Significantly improved insomnia post-treatment, with sustained reductions in anxiety and depression at both post-test and 3-month follow-up. Quality of life gains emerged by the 3-month follow-up
King et al. (2002)	United States	RCT	62.2 ± 9.3 (*n* = 45)	63.3 ± 9 (*n* = 40)	12 months	Physical exercise	Attention control	PSQI	Significant improvements in sleep quality in the intervention group relative to the control group.
Korn et al. (2009)	United States	RCT	<50: 6; >50: 15 (*n* = 21)	<50: 12; >50: 9 (*n* = 21)	8 sessions	Polarity therapy	Respite sessions	Overall sleep quality (PSQI)	Overall sleep quality and quality of life improved (nonsignificant between groups), while depression showed significant between-group improvement
Lee et al. (2007)	United Kingdom	Prospective case series	67.4 (34–87) (*n* = 39)		2 weeks	Institutional respite care	–	Sleep onset latency, total sleep time, WASO, sleep efficiency–(A); overall sleep quality (subjective sleep quality score); sleepiness (ESS)	Significantly increased total sleep time and improved overall sleep quality, but returned to baseline levels by follow-up. Sleep efficiency remained stable during respite but declined significantly at follow-up
Livingston et al. (2019)	United Kingdom	RCT	80.4 ± 9.0 (*n* = 42)	79.6 ± 7.0 (*n* = 20)	6 weeks	Behaviroal sleep intervention	Usual care	PSQI	Significant improvements on overall sleep quality
McCurry et al. (1998)	United States	RCT	Group IG: 68.0 ± 7.5 (*n* = 7) Individual IG: 64.9 ± 13.3 (*n* = 14)	72.6 ± 7.7 (*n* = 15)	6 weeks	Sleep education and caregiving skill training	Wait list	Sleep quality (PSQI); Sleep latency, sleep efficiency, WASO, sleep duration, number of night awakenings(A); overall sleep quality, felt upon awakening (sleep diary)	Overall sleep quality significantly improved post-test and at 3-month follow-up, with a trend toward lower depression scores. Sleep efficiency also improved significantly from Week 1 to Week 4, while other diary variables showed no significant changes
McCrae et al. (2023)	United States	Quasi-experimental study	62.4 ± 18.2 (*n* = 5)		4 weeks	Cognitive behavioral therapy for insomnia	–	WASO, sleep onset latency, sleep efficiency (daily electronic diaries); insomnia (ISI)	Significantly improved the sleep latency, WASO, sleep efficiency, insomnia, anxiety, depression, and quality of life at post-test
Nie et al. (2025)	China	RCT	42.58 ± 11.56 (*n* = 40)	45.53 ± 10.20 (*n* = 40)	8 weeks	Mindfulness-based stress reduction	Usual care	PSQI, actigraphy	Significantly improved sleep quality, sleep efficiency, and sleep duration, along with reduced sleep latency, fewer sleep disturbances, and less daytime dysfunction, but no significant difference was observed in the use of sleeping medication
Oken et al. (2010)	United States	Pilot RCT	62.50 ± 11.61 (*n* = 10)	67.09 ± 8.36 (*n* = 11)	7 weeks	Mindfulness meditation	Self-care education	Overall sleep quality (PSQI); sleepiness (ESS)	No significant differences on overall sleep quality, sleepiness, and depression between groups
Park et al. (2020)	South Korea	Quasi-experimental study	54.50 ± 3.71 (*n* = 12)	61.00 ± 6.42 (*n* = 12)	16 weeks	Caregiving skills training	Handbook	Sleep efficiency (self-report)	Failed to improve the sleep efficiency
Raldiris et al. (2020)	United States	RCT	*n* = 3	*n* = 6	2 weeks	Mindfulness training	Cognitive reappraisal training	Insomnia (ISI)	Both groups showed improvements in group means for insomnia
Rapaport et al. (2024)	United States	RCT	64. 6 ± 13.7 (*n* = 170)	63.5 ± 12.9 (*n* = 176)	6 sessions	Sleep education and caregiving skill training	Usual treatment	Insomnia (SCI)	Statistically improved the insomnia and anxiety at 8 months but failed to improve the depression
Rose et al. (2009)	United States	Pilot RCT	71.94 ± 7.78 (*n* = 19)	76.52 ± 5.60 (*n* = 19)	16 weeks	Cranial electrical stimulation (CES)	Sham CES	Overall sleep quality (PSQI); sleep disturbances (GSDS) sleep latency (sleep diary)	No significant differences in overall sleep quality or sleep disturbances, but a trend toward statistical significance in daily disturbances of the PSQI subscale and a decrease in sleep latency recorded by the sleep diary
Rowe et al. (2010)	United States	Quasi-experimental study	62 ± 11.90 (*n* = 45)		48 weeks	Installed Nighttime monitoring system at home	–	Sleep duration (A); number of awakenings, minutes awake during night, wake time, out-of-bed time, overall sleep quality (sleep diary)	No significant effects were found for group or interaction terms across all sleep measures.
Sakurai and Kohno (2020)	Japan	Quasi-experimental study	83.2 ± 11.8 (*n* = 10)		1 night	Institutional respite care	–	Total sleep time, sleep efficiency, sleep latency, WASA(A)	No significant differences in sleep time, efficiency, latency, or WASO between caregiving and respite days
Scheffer et al. (2025)	United States	RCT	63.03 ± 12.69 (*n* = 36)	66.18 ± 10.74 (*n* = 34)	9 months	Fully activated in-home assisted technology	Partially activated in-home assisted technology	PSQI	Caregivers in the control conditions reported significantly worsening sleep efficiency, whereas in comparison, those in the active conditions reported improving sleep efficiency
Shih et al. (2024)	China	Quasi-experimental design	77.1 ± 8.1 (*n* = 60)		24 weeks	Walking intervention	Usual activity	PSQI	A slight improvement in sleep quality of family caregivers, but not significant
Sloane et al. (2015)	United States	Crossover RCT	18–44: 3; 45–59: 7; ≥60: 7 (*n* = 17)		6 weeks	Light therapy	Crossover control	Overall sleep quality, sleep efficiency (PSQI); sleepiness (ESS); overall sleep problems (MOS)	Statistically improved the overall sleep quality and decreased the sleep problems but failed to improve the sleep efficiency and sleepiness
Simpson and Carter (2010)	United States	Quasi-experimental study	63 ± 14.85 (*n* = 6)		5 weeks	Sleep education + physical exercise	–	Overall sleep quality (PSQI); sleep efficiency, sleep duration, sleep latency, WASO(A);	The intervention enhanced the overall sleep quality, and depression trended toward improvement. But the changes did not reach statistical significance
Song et al. (2024)	United States	Pilot RCT	67.0 ± 10.9 (*n* = 30)		5 weeks	Sleep education + light explore + caregiving skill training	Sessions about general information about sleep, aging, and dementia	Overall sleep quality (PSQI); total awake time, sleep efficiency(A)	No significant trends in overall sleep quality and sleep efficiency from baseline to 3-month follow-up between the two groups
Tewary et al. (2018)	United States	Pre-post design	71.3 (*n* = 7)		6 weeks	Education and reinforcement on light exposure, exercise, and sleep hygiene	–	Sleep disorder inventory (SDI)	All seven participants had reduced frequency, severity, and distress on all seven factors measured through SDI
Williams et al. (2019)	United States	RCT	64.6 ± 12.2 (*n* = 56)	63.9 ± 13.7 (*n* = 41)	12 weeks	Caregiving skills training	Telephone-Support Attention Control	Overall sleep quality (PSQI)	No significant differences in overall sleep quality between groups, while depression showed significant between-group improvement
Xu et al. (2022)	China	Pilot RCT	54.71 ± 11.19 (*n* = 35)	53.44 ± 10.83 (*n* = 36)	8 weeks	Behavioral sleep intervention: Sleep education and caregiving skill training	Usual Care	Overall sleep quality (PSQI)	Significantly improved overall sleep quality, sleep efficiency, and depression according to the group-by-time interaction effects; only the sleep latency had a significant difference in the between-group effects

*Note.* A = actigraphy data; AD = Alzheimer disease; ADRD = Alzheimer disease and related dementia; CBT-I = cognitive behavioral therapy for insomnia; CG = control group; ESS = Epworth sleepiness scale; IG = intervention group; ISI = insomnia severity index; MOS = medical outcomes study sleep measures; PSQI = Pittsburgh sleep quality index; SCI = sleep conditions indicator; SDI = sleep domain of the neuropsychiatric inventory; WASO = wake after sleep onset.

### Risk of bias in included studies

The quality appraisal showed that the studies included were generally of moderate to low quality (Supplementary Files 1 and 2). Overall, 14 (46.7%) studies were appraised as having a “high risk” or “serious risk” of bias, and 13 (43.3%) studies were categorized as “some concerns” or “moderate risk.” Due to the difficulty of blinding participants in nonpharmacological treatments, common methodological limitations were the failure to apply blinding as well as the lack of transparency in randomized methods, the absence of analysis of missing data, and not evaluating the treatment effect using Intention-To-Treat (ITT) analysis (Supplementary Files 3 and 4 show the ranking of each evaluation item). Furthermore, explicit reporting of sample size justifications is often limited, evidence of sample representativeness is insufficient, and clarification of intervention types by the clinical guideline is lacking (Sidani et al., 2022).

### Types of nonpharmacological interventions used to improve caregivers’ sleep

Seven categories of interventions were classified: (1) multicomponent behavioral sleep intervention (*n* = 10); (2) sensory-based intervention (*n* = 7); (3) caregiver education (*n* = 5); (4) physical exercise (*n* = 3); (5) institutional respite care (*n* = 2); (6) cranial electrical stimulation (*n* = 1); and (7) nighttime monitoring system (*n* = 2). Multicomponent behavioral sleep interventions generally included components on sleep education and caregiving skills training. Sensory-based interventions included light therapy, mindfulness, and massage. Caregiver education included resources on the background of dementia, caregiving skills, and self-care techniques. Details are shown in Supplementary File 5.

### Effect of nonpharmacological interventions on caregivers’ sleep outcomes

#### Effects of nonpharmacological interventions on overall sleep quality

**Figure 2:**
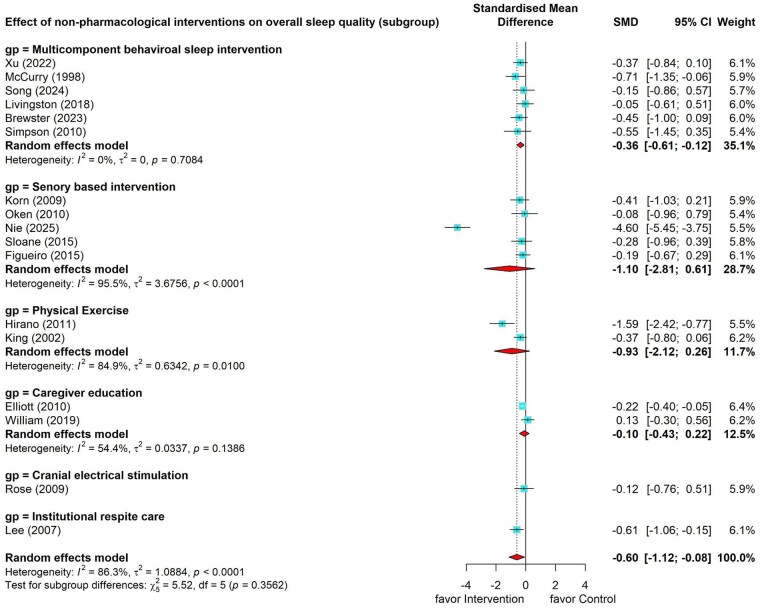
Effects of nonpharmacological interventions on overall sleep quality.

The meta-analysis of 17 studies measuring the effect on overall sleep quality showed that nonpharmacological interventions had a moderate pooled effect on subjective sleep quality (SMD = –0.60, 95% CI = –1.12 to –0.08) (Brewster et al., 2023; Elliott et al., 2010; Figueiro et al., 2015; Hirano et al., 2011; King et al., 2002; Korn et al., 2009; Lee et al., 2007; Livingston et al., 2019; McCurry et al., 1998; Nie et al., 2025; Oken et al., 2010; Rose et al., 2009; Simpson & Carter, 2010; Sloane et al., 2015; Song et al., 2024; Williams et al., 2019; Xu et al., 2022). However, substantial heterogeneity existed (*I*^2^ = 86.30%, *p* < .001), and subsequent subgroup analyses were conducted (Figure 2). Through sensitivity analysis by excluding nonrandomized controlled studies, the results showed a similar effect size, but it turned out to be insignificant for all the overall nonpharmacological interventions (Supplementary File 6). However, the multicomponent behavioral sleep intervention subgroup remained significant, and the effect of behavioral sleep intervention was robust.

##### Effect of multicomponent behavioral sleep intervention on overall sleep quality

Subgroup analysis showed that six multicomponent behavioral sleep interventions had a small pooled effect on improving overall sleep quality immediately post-intervention (SMD = –0.36, 95% CI = –0.61 to –0.12) (Brewster et al., 2023; Livingston et al., 2019; McCurry et al., 1998; Simpson & Carter, 2010; Song et al., 2024; Xu et al., 2022), while the results suggest no statistical heterogeneity (*I*^2^ = 0, *p* = .71; Figure 2).

##### Effect of sensory-based intervention on overall sleep quality

Five studies (Figueiro et al., 2015; Korn et al., 2009; Nie et al., 2025; Oken et al., 2010; Sloane et al., 2015) evaluating the effects of sensory-based intervention showed large but nonsignificant improvement in caregivers’ overall sleep quality, with large heterogeneity (SMD = –1.10, 95% CI = –2.81 to 0.61; *I*^2^ = 95.5%, *p* < .001; Figure 2).

##### Effect of other interventions on overall sleep quality

The effects of two studies using caregiver education had conflicting results regarding the effect on caregivers’ overall sleep quality: one showed a small significant effect size (Hedges’ *g* = –0.22, 95% CI = –0.40 to –0.05) (Elliott et al., 2010), while the other showed no significant effect (Hedges’ *g* = 0.13, 95% CI = –0.30 to 0.56) (Williams et al., 2019). The caregivers that accepted institutional respite care reported a significant improvement in their overall sleep quality (Hedges’ *g* = –0.61, 95% CI = –1.06 to –0.15) (Lee et al., 2007). The effects of physical exercise showed a large but insignificant effect on overall sleep quality (SMD = –0.93, 95% CI = –2.12 to 0.26) (Hirano et al., 2011; King et al., 2002).

#### Effects of nonpharmacological interventions on sleep efficiency

**Figure 3:**
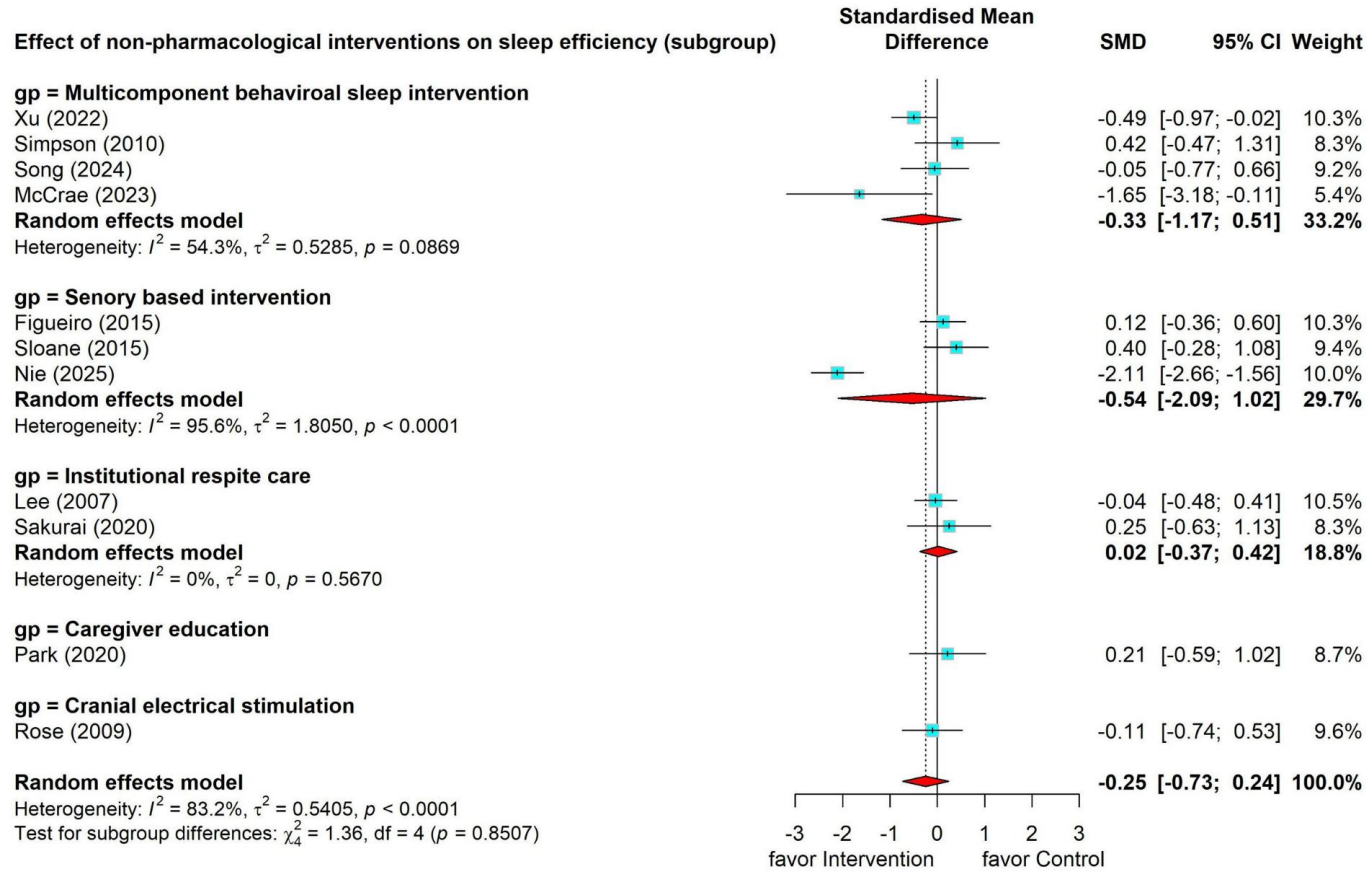
Effects of nonpharmacological interventions on sleep efficiency.

Eleven studies (Figueiro et al., 2015; Lee et al., 2007; McCrae et al., 2023; Nie et al., 2025; Park et al., 2020; Rose et al., 2009; Sakurai & Kohno, 2020; Simpson & Carter, 2010; Sloane et al., 2015; Song et al., 2024; Xu et al., 2022) examined the effects of nonpharmacological interventions on caregivers’ sleep efficiency, with six of them using actigraphy as the measurement tool (Figueiro et al., 2015; Nie et al., 2025; Sakurai & Kohno, 2020; Simpson & Carter, 2010; Song et al., 2024). The meta-analysis showed a small and nonsignificant pooled effect (SMD = –0.25, 95% CI –0.73 to 0.24), with substantial heterogeneity (*I*^2^ = 83.2%, *p* < .001). Subgroup analysis was conducted. The results were presented in Figure 3.

##### Effects of multicomponent behavioral sleep intervention on sleep efficiency

The subgroup analysis on multicomponent behavioral sleep intervention showed a small but still nonsignificant effect on sleep efficiency (SMD = –0.33, 95% CI = –1.17 to 0.51) with moderate heterogeneity (*I*^2^ = 54.3%, *p* = .087) (McCrae et al., 2023; Simpson & Carter, 2010; Song et al., 2024; Xu et al., 2022) (Figure 3).

##### Effects of other interventions on sleep efficiency

Sensory-based intervention demonstrated mixed results, with mindfulness training showing a large significant effect size (Hedges’ *g* = –2.11, 95% CI = –2.66 to –1.56) (Nie et al., 2025), while light therapy showed small insignificant effects (Hedges’ *g* = –0.12, 95% CI = –0.36 to 0.60; Hedges’ *g* = 0.40, 95% CI = –0.28 to 1.08) (Figueiro et al., 2015; Sloane et al., 2015). Institutional respite care showed nonsignificant small (Hedges’ *g* = –0.04, 95% CI = –0.48 to 0.41) (Lee et al., 2007) to small (Hedges’ *g* = 0.25, 95% CI = –0.63 to 1.13) (Sakurai & Kohno, 2020) effects on sleep efficiency. Caregiver education (Hedges’ *g* = 0.21, 95% CI = –0.59 to 1.02) (Park et al., 2020) and cranial electrical stimulation (Hedges’ *g* = –0.11, 95% CI = –0.73 to 0.24) had a nonsignificant effect (Rose et al., 2009) (Figure 3).

#### Effects of nonpharmacological interventions on sleep latency

**Figure 4:**
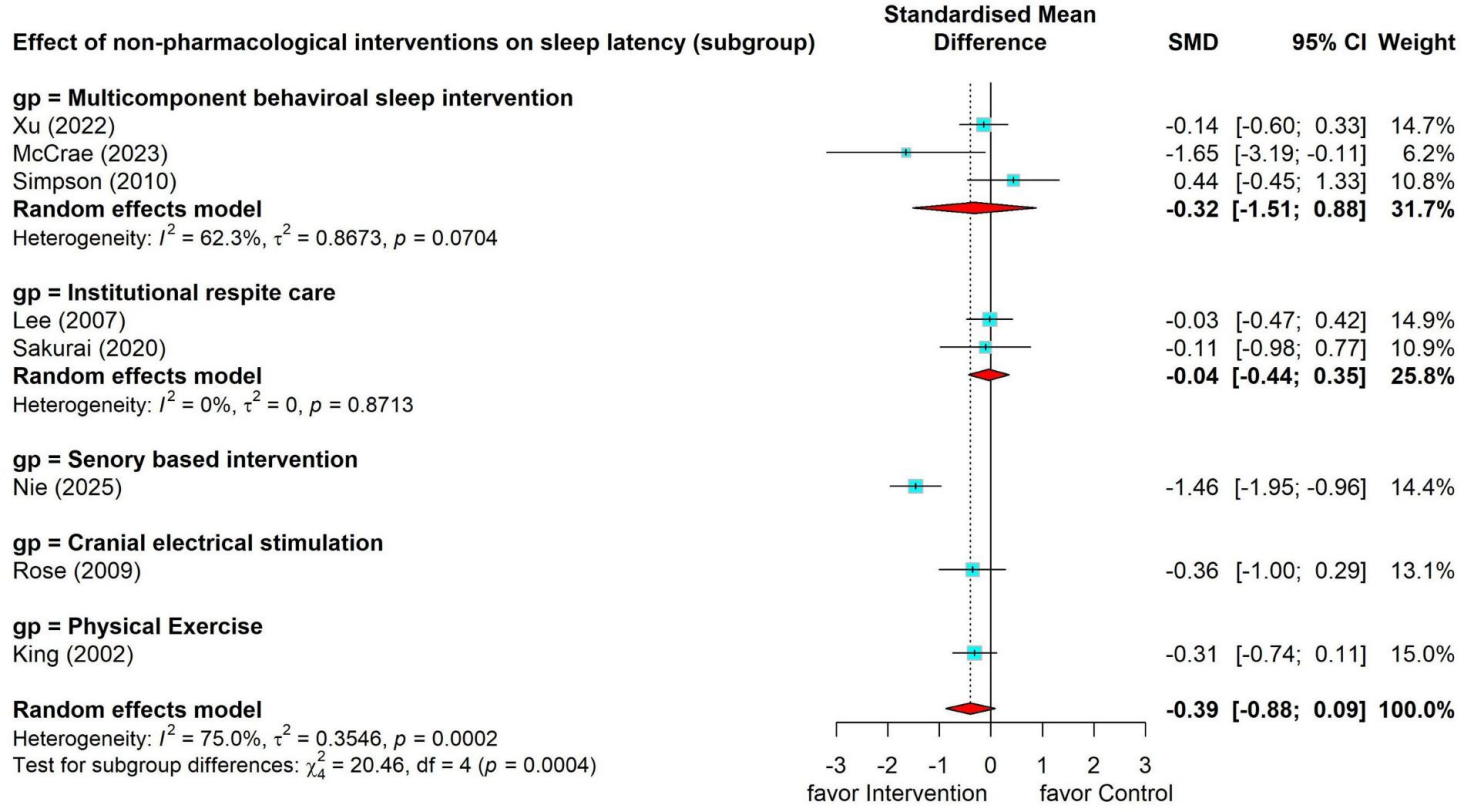
Effects of nonpharmacological interventions on sleep latency.

Eight studies measured the effects of nonpharmacological interventions on sleep latency (King et al., 2002; Lee et al., 2007; McCrae et al., 2023; Nie et al., 2025; Rose et al., 2009; Sakurai & Kohno, 2020; Simpson & Carter, 2010; Xu et al., 2022), and the result showed a small nonsignificant pooled effect (SMD = –0.39, 95% CI = –0.88 to 0.09), with substantial heterogeneity (*I*^2^ = 75.0%, *p* < .001; Figure 4). Subgroup analysis of multi-component behavioral sleep interventions showed a small but nonsignificant effect (SMD = –0.32, 95% CI = –1.51 to 0.88). Effect sizes for the two studies on institutional respite care showed small, nonsignificant effects on sleep latency (Hedges’ *g* = –0.03 to –0.11) (Lee et al., 2007; Sakurai & Kohno, 2020). One individual study on sensory-based intervention showed large significant effects (Hedges’ *g* = –1.46, 95% CI = –1.95 to –0.96) (Nie et al., 2025), while another two individual studies representing cranial electrical stimulation (Rose et al., 2009) and physical exercise (King et al., 2002) showed small insignificant effects on sleep latency (Hedges’ *g* = –0.36 to –0.31).

#### Effects of nonpharmacological interventions on caregivers’ sleep duration

**Figure 5:**
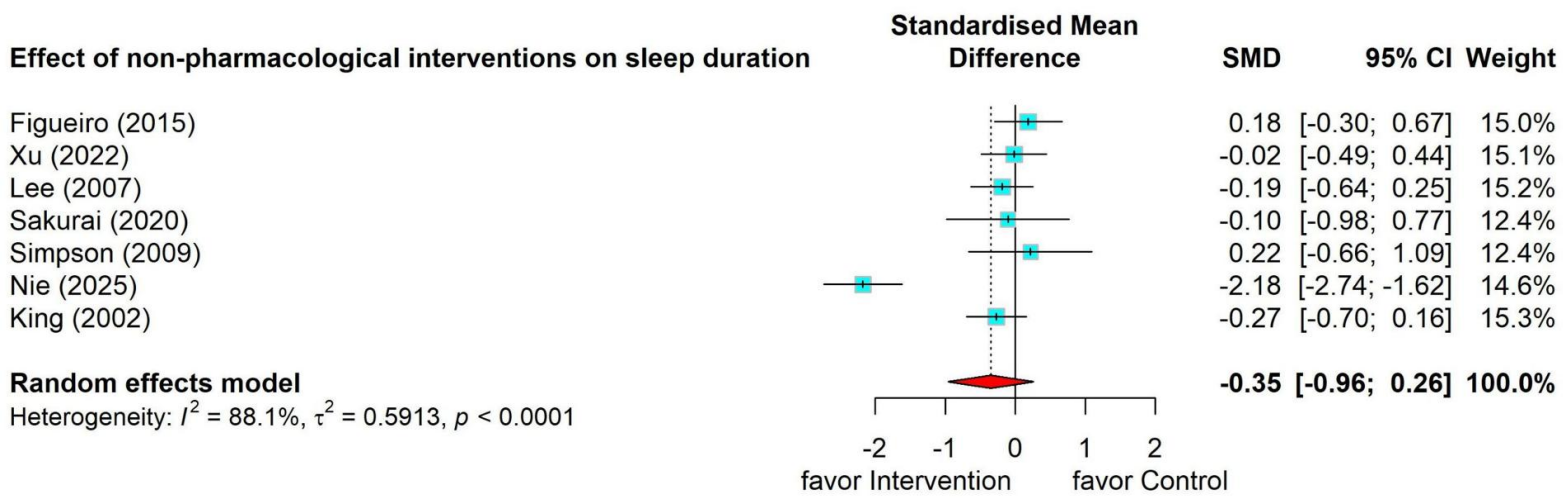
Effects of nonpharmacological interventions on sleep duration.

The meta-analysis of seven studies (Figueiro et al., 2015; King et al., 2002; Lee et al., 2007; Nie et al., 2025; Sakurai & Kohno, 2020; Simpson & Carter, 2010; Xu et al., 2022) testing the effects of nonpharmacological interventions on sleep duration revealed a small and nonsignificant pooled effect (SMD = –0.35, 95% CI = –0.96 to 0.26), with substantial heterogeneity (*I*^2^ = 88.1%, *p* < .001). Subgroup analysis was not conducted due to the limited number of studies in each subgroup (Figure 5).

#### Effects of nonpharmacological interventions on wake after sleep onset

**Figure 6:**
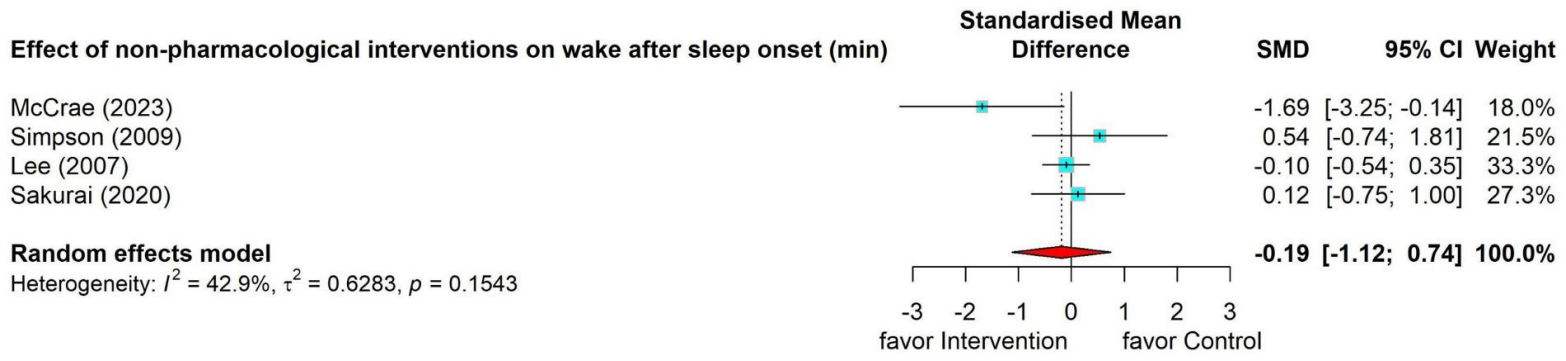
Effects of nonpharmacological interventions on wake after sleep onset.

Four studies (Lee et al., 2007; McCrae et al., 2023; Sakurai & Kohno, 2020; Simpson & Carter, 2010) revealed a small and nonsignificant pooled effect (SMD = –0.19, 95% CI = –1.12 to 0.74) on wake after sleep onset, with moderate heterogeneity (*I*^2^ = 42.9%, *p* = .15). Subgroup analysis was not conducted due to the limited number of studies in each subgroup (Figure 6).

#### Effects of nonpharmacological interventions on insomnia

**Figure 7:**
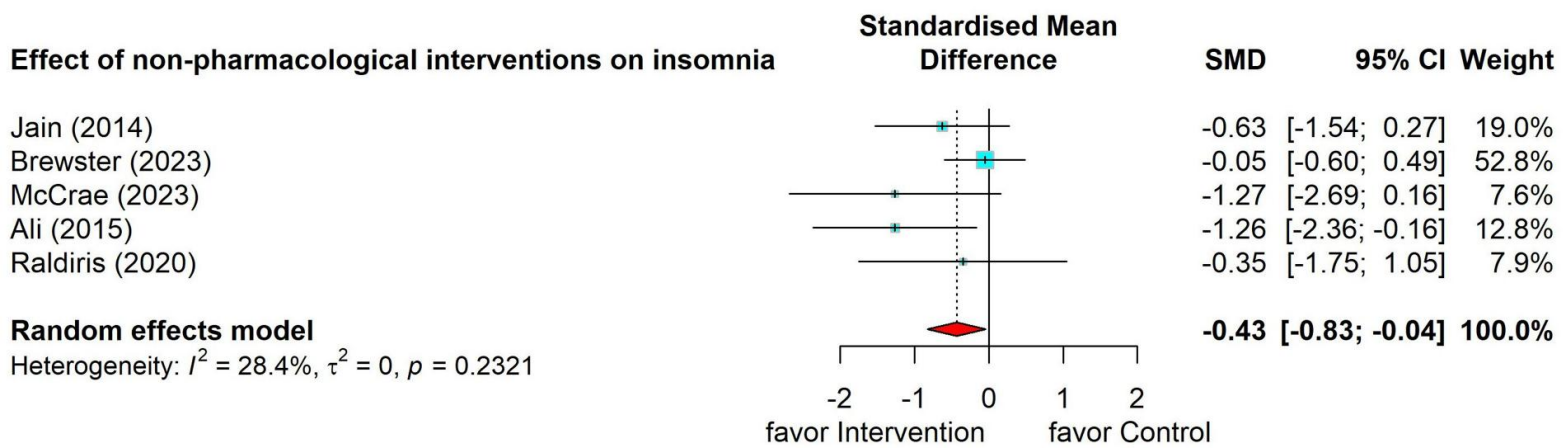
Effects of nonpharmacological interventions on insomnia.

Five studies (Ali & Bokharey, 2015; Brewster et al., 2023; Jain et al., 2014; McCrae et al., 2023; Raldiris, 2020) tested the effect of nonpharmacological interventions in improving insomnia symptoms among caregivers and showed a small significant effect (SMD = –0.43, 95% CI = –0.83 to –0.04; Figure 7).

### Subgroup analysis based on subjective versus objective measures

The outcomes of sleep quality and insomnia were all measured in a subjective way. Therefore, we conducted subgroup analysis for sleep latency, WASO, and sleep duration. For WASO, although subjective (SMD = –1.69, 95% CI = –3.25 to 0.14), it tended to be significantly better than objective (SMD = 0, 95% CI = –0.38 to 0.38), but there is only one study with subjective WASO measures, so the result may not be convincing. Basically, there is no significant difference between objective versus subjective measures (Supplementary Files 7–9).

## Discussion

To the best of our knowledge, this systematic review represents the first attempt to synthesize the types of nonpharmacological interventions designed to improve sleep among informal caregivers of people with dementia and to evaluate their effects through meta-analysis and appropriate subgroup analyses. This review contributes to the field by systematically analyzing existing evidence on nonpharmacological interventions for addressing sleep problems in this population, which, despite being highly prevalent, remains underexplored. Seven distinct types of interventions, comprising diverse components and reported in 30 studies, were identified, highlighting the heterogeneity of implemented interventions. These findings underscore a significant research gap that requires further investigation to inform best practices and guide future research.

The significant moderate pooled effect of nonpharmacological interventions on the overall subjective sleep quality underscores the potential of such interventions in enhancing sleep among informal caregivers of people with dementia. Subgroup and sensitivity analyses further revealed the significant effects of multicomponent behavioral sleep interventions, demonstrating the greatest efficacy among all nonpharmacological approaches. This may be attributed to the fact that these multicomponent interventions were predominantly grounded in cognitive behavioral therapy for insomnia (CBT-I) principles, the gold standard for improving sleep in the general population, and incorporated both strategies for modifying sleep behaviors and training in caregiving skills. Comparatively, subgroup analysis of standalone education on caregiving skills (Elliott et al., 2010; Williams et al., 2019) or sensory-based interventions (Figueiro et al., 2015; Korn et al., 2009; Nie et al., 2025; Oken et al., 2010; Sloane et al., 2015), which have been effective in improving sleep among the general population (Chan et al., 2022; Fang et al., 2023), exhibited no significant effects on sleep quality. A similar significant finding was identified on improving insomnia. These findings emphasize the importance of comprehensive training rooted in CBT-I principles and the critical need to address the unique stressors associated with caregiving that cause sleep problems in caregivers. Multicomponent behavioral sleep interventions effectively incorporated these aspects by changing dysfunctional beliefs and attitudes about caregiving and sleep through cognitive restructuring, sleep hygiene education, stimulus control, sleep restriction, and relaxation techniques (Altena, 2022; Wang et al., 2021). Even though promising, due to the high heterogeneity and the diversity of regions, the findings still need to be interpreted with caution. A bigger sample and rigorously designed research are suggested to further validate the effects of multicomponent behavioral sleep interventions.

Overall, a limited number of studies have examined the effects of nonpharmacological interventions on specific sleep parameters. Among the 11 studies reporting on sleep efficiency, a small and statistically nonsignificant pooled effect was identified, suggesting that, as a whole, these interventions may not significantly enhance sleep efficiency for informal caregivers of people with dementia. This may be due to substantial heterogeneity among studies regarding intervention components and measurement tools. The subgroup analysis revealed that multicomponent behavioral sleep interventions have a mild to moderate but statistically nonsignificant effect. Since these interventions provide training on sleep behavior change, the nonsignificant effect could be due to the fact that all included studies were pilot studies with small sample sizes. These findings align with reviews in other populations, such as people with dementia (Wilfling et al., 2023) and insomnia disorders (Chambe et al., 2023), which consistently indicate only a trend toward improvement in sleep efficiency through nonpharmacological interventions, with further research required to confirm these effects. Behavioral change is a gradual process, and establishing new habits takes time to show significant effects (Verplanken & Orbell, 2022). The included studies only had short durations of four to eight weeks, which may not be sufficient to observe significant changes in sleep efficiency. While some studies suggest that light therapy may improve sleep efficiency by modulating circadian rhythms (LeGates et al., 2014), only two studies in this review used light therapy, showing different effects (Figueiro et al., 2015; Sloane et al., 2015). More research with large sample sizes is needed to examine the effects of nonpharmacological interventions on sleep efficiency.

The meta-analysis on other sleep parameters did not reveal significant pooled effects, similar to findings on sleep efficiency. However, multicomponent behavioral sleep interventions showed the most promising effects on sleep latency and wake after sleep onset. Notably, the study co-designed with caregivers, which combined sleep education with caregiving skill training and utilized a flexible online platform, demonstrated the largest effect size (McCrae et al., 2023). This highlights the importance of co-designing interventions to tailor components and ensure flexibility in delivery, addressing the unique needs and demanding tasks of caregiving (Wang et al., 2021). Surprisingly, institutional respite care did not show promising effects, despite being considered an essential component of home care (Utz, 2022). It may be because transitioning a loved one into respite care can be emotionally challenging for caregivers. Concerns about the quality of care provided and feelings of abandoning their loved ones during the respite period can potentially increase caregivers’ anxiety and stress rather than alleviating it (Leocadie et al., 2018). Additionally, it is also unlikely that a caregiver experiencing disrupted sleep will see a dramatic improvement in sleep quality during a brief respite break, as learned nocturnal hypervigilance can take a long time to dissipate (McCurry et al., 2007). Therefore, the temporary nature of respite care may not be sufficient for caregivers to relax and recover from chronic sleep deprivation. This finding aligns with another review that indicated mixed effects of temporary residential respite (Vandepitte et al., 2016) and an empirical study showing that respite care only influenced the first half of the sleep period (Sakurai & Kohno, 2020). These findings underscore the persistent impact of caregiving on sleep and the complexity of factors influencing caregivers’ sleep. More efforts are needed to enhance caregivers’ sleep by developing and implementing more effective interventions.

### Implications for nursing and health policy

The findings of this review underscore the importance of implementing evidence-based, nonpharmacological interventions to improve sleep quality among informal caregivers of people with dementia. In particular, multicomponent behavioral sleep interventions, especially those incorporating CBT-I principles and caregiving skills training, are essential for nurses and healthcare professionals to use in supporting caregivers. Theory-driven approaches should be prioritized over standalone education or exercise.

From a health policy perspective, it is essential to develop and promote structured programs that target both sleep behavior modification and the unique challenges faced by caregivers. Policymakers should consider prioritizing resource allocation to the implementation of multicomponent behavioral interventions in both community and clinical settings. Moreover, further investment in large-scale, methodologically robust trials is necessary to build a stronger evidence base for improving sleep disturbances among caregivers.

### Limitations and implications

Despite the findings of this meta-analysis, several limitations need to be taken into account. Most of the studies included were with small sample sizes, which may limit the validity of the results. Therefore, more high-quality, large-scale studies are recommended. Although subgroup analysis supported the robustness of the estimates, the evidence remains insufficient to draw firm conclusions due to the limited number of studies and substantial heterogeneity. Even though we made efforts to reduce heterogeneity through subgroup and sensitivity analyses, the influence of other potential sources, such as the region, cultural elements, variations in implementation fidelity, and differences among participants, cannot be overlooked. Moreover, we were unable to investigate several potentially important moderators of treatment effects, such as caregiver age, care recipient disease duration and dementia severity, and key intervention characteristics (e.g., intervention dosage and the interval between pre- and post-intervention assessments). Future trials are recommended to systematically report and examine these factors. Additionally, most studies did not report effects on specific sleep parameters and lacked the use of objective measures for sleep. As a result, the findings are mainly confined to caregivers’ perceived sleep quality. Because subjective assessments can be influenced by expectancy effects, social desirability, and the caregiver’s overall psychological state, the predominance of self-report data may overstate perceived improvements relative to objective sleep change. Combining standardized subjective instruments with objective metrics (actigraphy or polysomnography) is suggested for future research to reduce measurement bias and provide a fuller picture of intervention effects. Given the limited number of studies for each type of nonpharmacological intervention and the preliminary nature of the evidence base, a formal GRADE assessment was not employed to formally rate the certainty of evidence. Another limitation is that only a small portion of trials reported follow‑up outcomes beyond the immediate post‑intervention assessment. As a result, our conclusions primarily reflect short‑term effects on sleep, and the sustainability of these benefits remains unclear. Future trials should incorporate longer follow‑up periods to evaluate the long‑term effectiveness and sustainability of nonpharmacological sleep interventions for dementia caregivers. Furthermore, while our inter-rater reliability indicated substantial agreement (Landis & Koch, 1977), future reviews could improve this metric by implementing formal pilot-calibration exercises prior to the screening phase. Due to the limited number of studies for each type of control, we were unable to perform a subgroup analysis. The inclusion of various control groups, especially when the majority were not active controls, may overestimate the actual effect size to some extent. In addition, we did not include unpublished or grey literature (e.g., conference abstracts), which may limit the comprehensiveness of the evidence base and could contribute to publication bias. However, these sources typically provide insufficient detail on intervention content, study quality, and outcome data for meaningful risk of bias assessment and meta-analytic synthesis. Finally, all the included studies are published in English. This language restriction may have introduced a risk of language bias.

## Conclusions

This systematic review and meta-analysis indicate that nonpharmacological interventions, particularly multicomponent behavioral sleep interventions, have significant effects on the overall sleep quality of informal caregivers of people with dementia. A significant pooled effect on insomnia was also observed. However, the effects on specific sleep parameters remain limited, largely due to the small sample sizes and the limited number of studies included. These findings emphasize the urgent need for high-quality, large-scale clinical trials to validate the results. Additionally, incorporating objective sleep measures is recommended to provide a more accurate assessment of sleep improvements. Future research is suggested to focus on targeted and individualized approaches that address the unique challenges and specific sleep needs of caregivers of people with dementia.

## Supplementary Material

gnag081_Supplementary_Data

## Data Availability

The data, analytic methods, or materials are available from the corresponding author on reasonable request. This review has been pre-registered in PROSPERO (ref: CRD42024594090).
