# Supplementary material for: Nonpharmacological interventions to improve the sleep of informal caregivers of people with dementia: a systematic review and meta-analysis
Source: Gerontologist. 2026 Apr 29;66(6):gnag081. doi: 10.1093/geront/gnag081 (PMC13221722; doi:10.1093/geront/gnag081)

Non-pharmacological interventions to improve the sleep of informal caregivers of people with dementia: A systematic review and meta-analysis

Shanshan Wang, Yuqian Luo, Jiaming Xiong, Yuting Xia, Sze Him Isaac Leung, Patricia M. Davidson

Supplementary File 1 Quality assessment of included studies using the Cochrane Risk of Bias tool (ROB2).

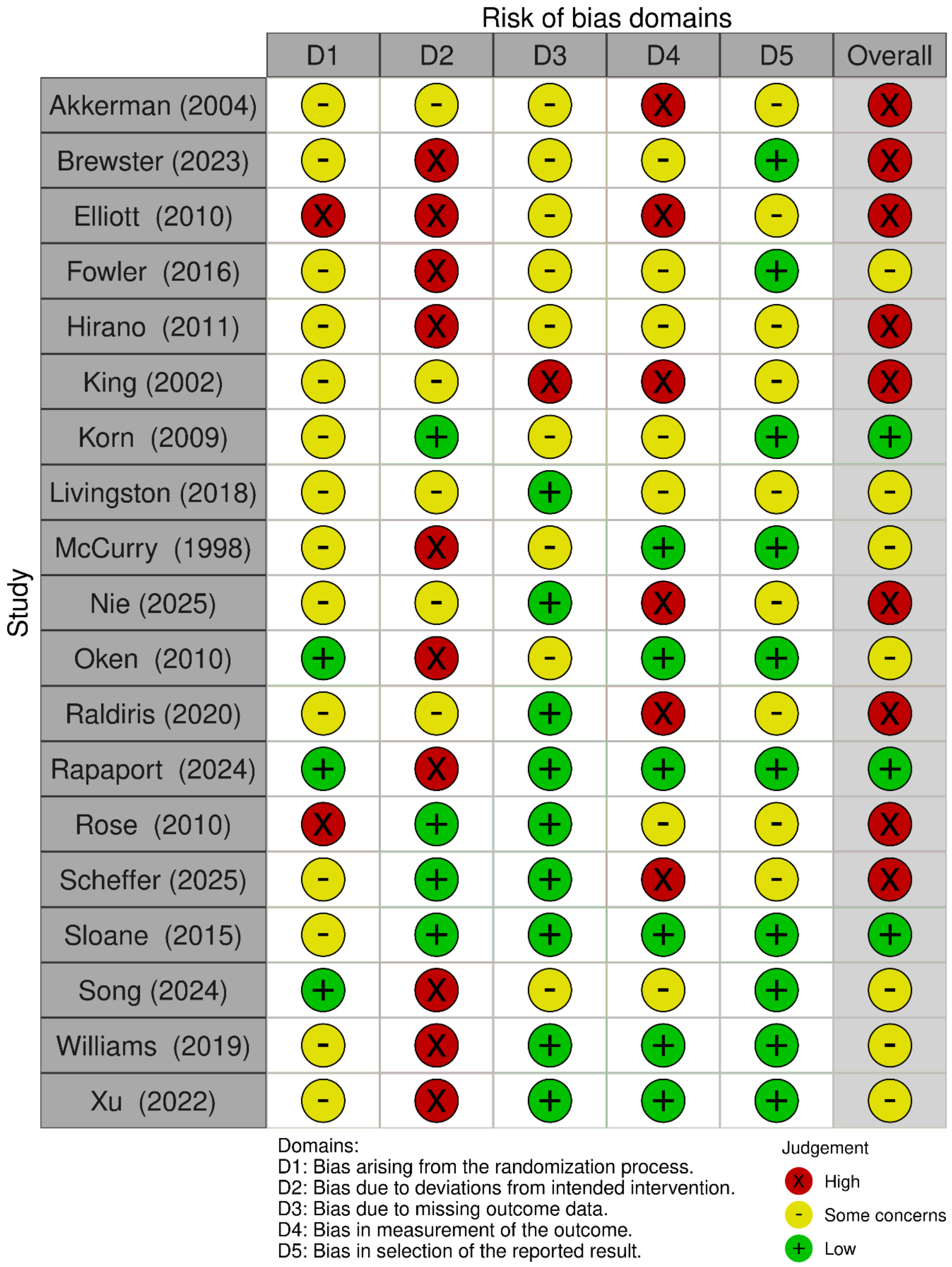

Supplementary File 2 Quality assessment of included studies using the Risk Of Bias In Nonrandomized Studies-of Interventions tool (ROBINS-I v2).

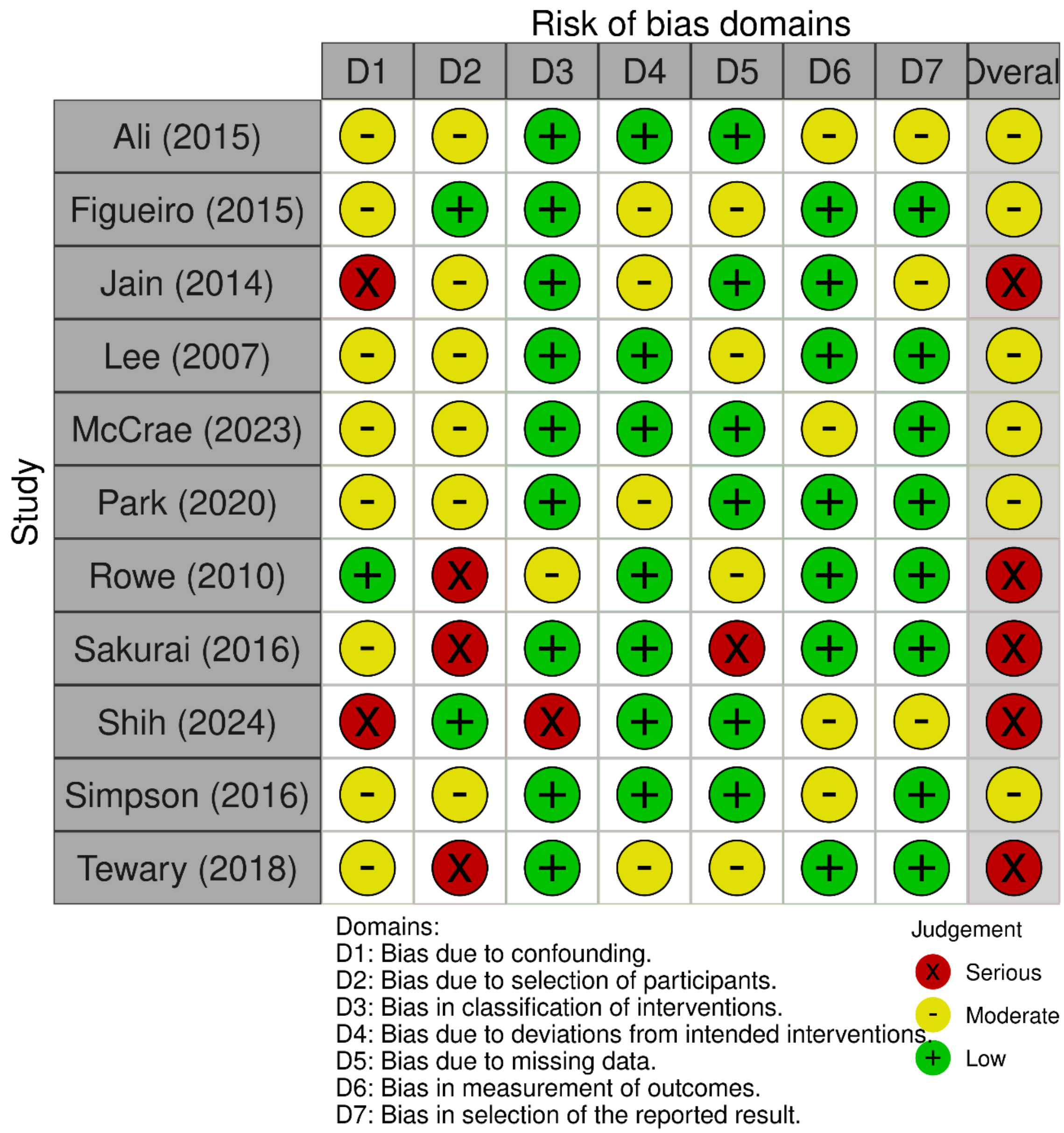

Supplementary File 3 Summary plot of quality assessment in studies using the Cochrane Risk of Bias tool (ROB2).

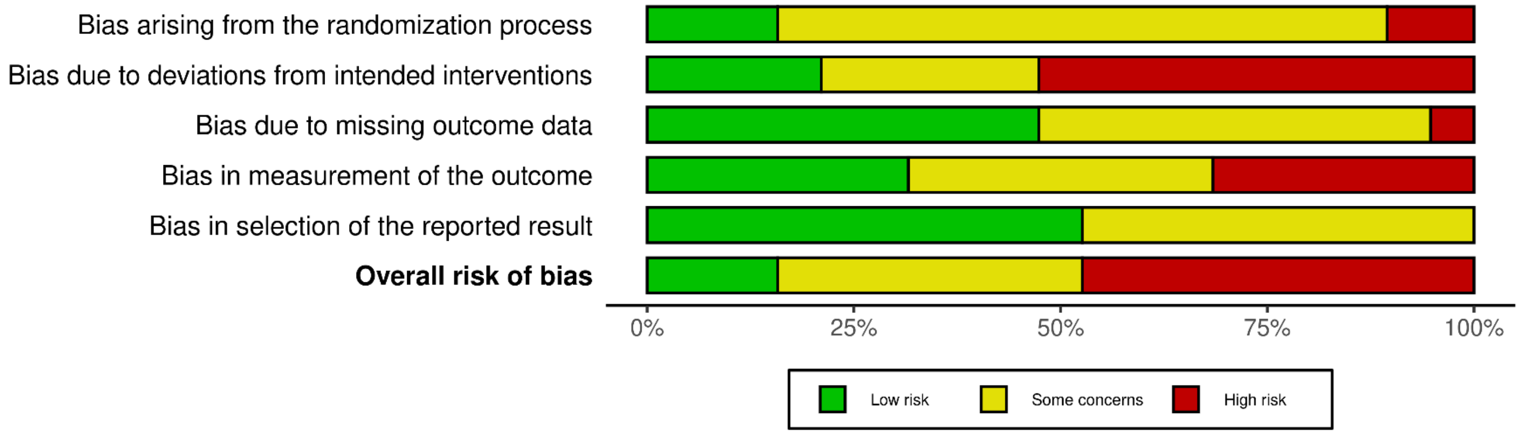

Supplementary File 4 Summary plot of quality assessment in studies using the Risk Of Bias In Nonrandomized Studies-of Interventions tool (ROBINS-I v2).

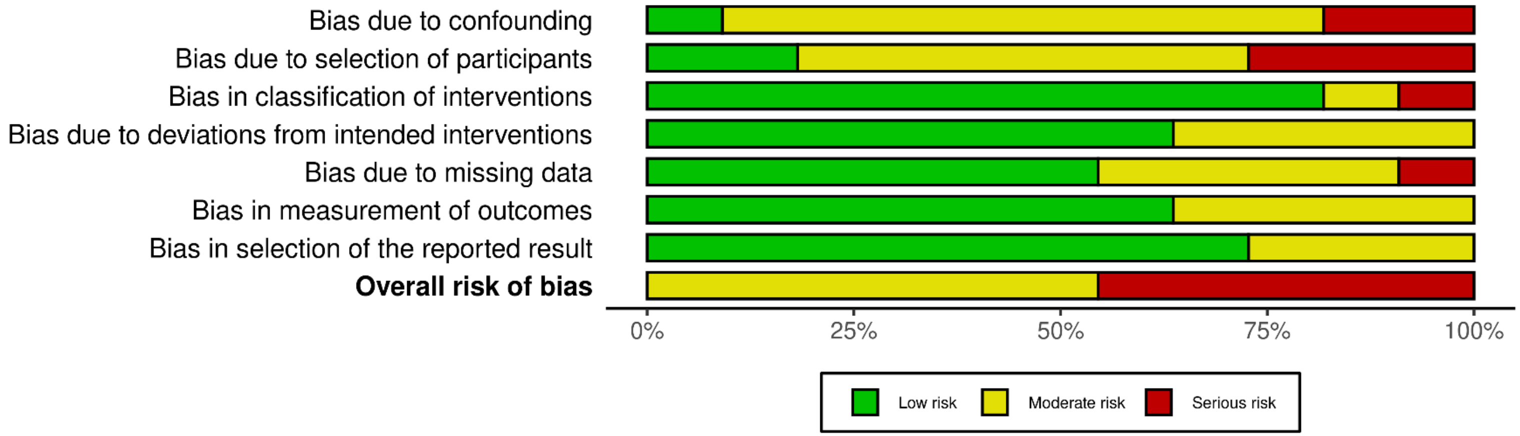

Supplementary File 5 The details of each type of non-pharmacological intervention

Types	Sub types	Details of interventions	Measurement Tool
Sensory based intervention:	Light therapy	Home setting tailored daytime lighting intervention. (Figueiro et al., 2015)	Sleep efficiency(A); Sleep duration(A);
		Home setting Environmental light during individuals' normal waking hours.(Sloane et al., 2015)	Sleep efficiency (PSQI); PSQI; Sleep Problems; Sleepiness;
	Mindfulness meditation	Eight sections of mindfulness-based interventions (meditation and guided Imagery training)(Jain et al., 2014)	Insomnia;
		6 weekly 90-min group sessions: Mindfulness meditation (Oken et al., 2010)	PSQI; Sleepiness
		Fourteen individual sessions in 2 weeks: mindfulness training ("Monitor+Accept") (Raldiris, 2020)	Insomnia
		8 weekly sessions of mindfulness-based stress reduction (Nie et al., 2025)	PSQI
	Massage	8 50-min massage sessions: Polarity therapy, applying manual pressure (both static and pulsating) on soft tissue pressure points. (Korn et al., 2009)	PSQI
Physical exercise		Home setting regular exercise with moderate intensity (3METs) 3 times per week.(Hirano et al., 2011)	Sleep quality (self-report)
		24 weeks, at least 120 minutes of walking per week (30 minutes per day, four times per week) with family caregiver or care attendant (Shih et al., 2024)	PSQI
		12-month home-based exercise (brisk walking + leisurely walking and gardening) (King et al., 2002)	PSQI

Institutional respite care		2 weeks of institutional respite care (Lee et al., 2007)	Sleep onset latency(A); Total sleep time(A); WASO(A); Sleep efficiency(A);
		1-night institutional respite care (Sakurai & Kohno, 2020)	Total sleep time(A); Sleep efficiency(A); Sleep latency(A); WASA(A)
Cranial Electrical Stimulation		Wear the Cranial Electrical Stimulation (CES) device for 60 min/day for 4-week. (Rose et al., 2009)	Daily disturbances (PSQI); overall PSQI
Caregiver education	Caregiving and self-care training	6 months 12 sessions (9 in home and 3 telephone sessions) and 5 structured telephone support group sessions.) Caregiving skill training (Knowledge of diseases + self-care knowledge + care knowledge and skills + relaxation techniques). (Elliott et al., 2010)	Sleep quality (self-report)
		4 months website usage: Caregiving skill training (Knowledge of diseases + care knowledge and skills + self-care knowledge + peer interaction).(Fowler et al., 2016)	Insomnia; Sleep quality (A)
		7 weekly email 75-90min group video conferences Caregiving skill training: (Knowledge of diseases + care knowledge and skills + self-care knowledge). (Brewster et al., 2023)	/
	Caregiving skills training	4 weeks of knowledge library in a mobile app: Caregiving skill training (Knowledge of diseases + care knowledge and skills).(Park et al., 2020)	Sleep efficiency
		3 months of weekly tailored interventions through group discussion: Caregiving skill training (multidisciplinary professional guidance about care knowledge and skills). (Williams et al., 2019)	PSQI;
Multicomponent	Sleep	6 weeks sessions: Sleep education (sleep hygiene + sleep	PSQI;

behavioral sleep intervention	education and caregiving skill training	compression + stimulus control + relaxation techniques) + Caregiving skill training (care skills counseling). (McCurry et al., 1998)	
		8 weekly individual telephone-based sessions of specific BA techniques on Sleep hygiene and Caregiving skill training. (Xu et al., 2022)	PSQI;
		4 weekly session web-based Sleep education (sleep education + sleep hygiene + stimulus control + sleep compression + relaxation) + Caregiving skill training (techniques for caregivers) (McCrae et al., 2023)	Insomnia; WASO, sleep onset latency, sleep efficiency (Daily electronic diaries) ;
		6 weekly or fortnightly Sleep education (sleep hygiene+ stimulus control)+ Caregiving skill training (Knowledge of diseases + care knowledge and skills)+ daytime behavioral activation(maintain alertness and reduce naps).(Rapaport et al., 2024)	/
	Sleep education + light exposure + caregiving skill training	5 weekly 1h sessions: Sleep education (sleep hygiene + sleep compression + stimulus control+ relaxation techniques) + goal for nature light exposure + Physical exercise (goals for walking) + Caregiving skill training (care knowledge and skills). (Song et al., 2024)	PSQI; sleep efficiency(A); total wake time
		6 sessions over a 3-month period: psychoeducation + light therapy + establishing a new sleep-wake schedule + behavioral activation + relaxation + coping skills for families (Livingston et al., 2019)	PSQI

	Cognitive behavioral therapy + psychoeducation	10 sessions over 5-8 weeks: sleep education (cognitive restructure + relaxation training + sleep hygiene) + emotion management (psychoeducation + anger management + stress management + subjective burden management) (Ali & Bokharey, 2015)	Insomnia (a subscale in General Health Questionnaire-28)
		9 weekly meetings: didactic skill training + multidimensional model for caregiver anxiety (Akkerman & Ostwald, 2004)	Total awake minutes, wake episodes, duration of wake episodes (A)
	Sleep education + physical exercise	5 weekly 60-90min sessions: Sleep education (Sleep hygiene + stimulus control + relaxation) + Goal for physical exercise.(Simpson & Carter, 2010)	PSQI; Sleep efficiency(A); Sleep duration(A); Sleep latency(A); Wake after sleep onset(A)
	Sleep education + light exposure + physical exercise	Six one-hour in-home sessions over a two months period regarding dementias education and reinforcement on light exposure, exercise, and sleep hygiene (Tewary et al., 2018)	Sleep Disorder Inventory
Nighttime Monitoring System		Deploy a device which alerts the caregiver when the care-recipient leaves their bed at night. (Rowe et al., 2010)	Sleep duration(A); Number of awakenings, minutes awake during night, wake time, out-of-bed time, sleep quality(self-report)

In-home assistive technology (PPCg system) (Scheffer et al., 2025)	PSQI

Note: A: Actigraphy data; PSQI: Pittsburgh Sleep Quality Index; WASO: Wake after sleep onset; METs: metabolic equivalents.

Supplementary File 6 Sensitivity results excluding non-randomized studies on overall sleep quality

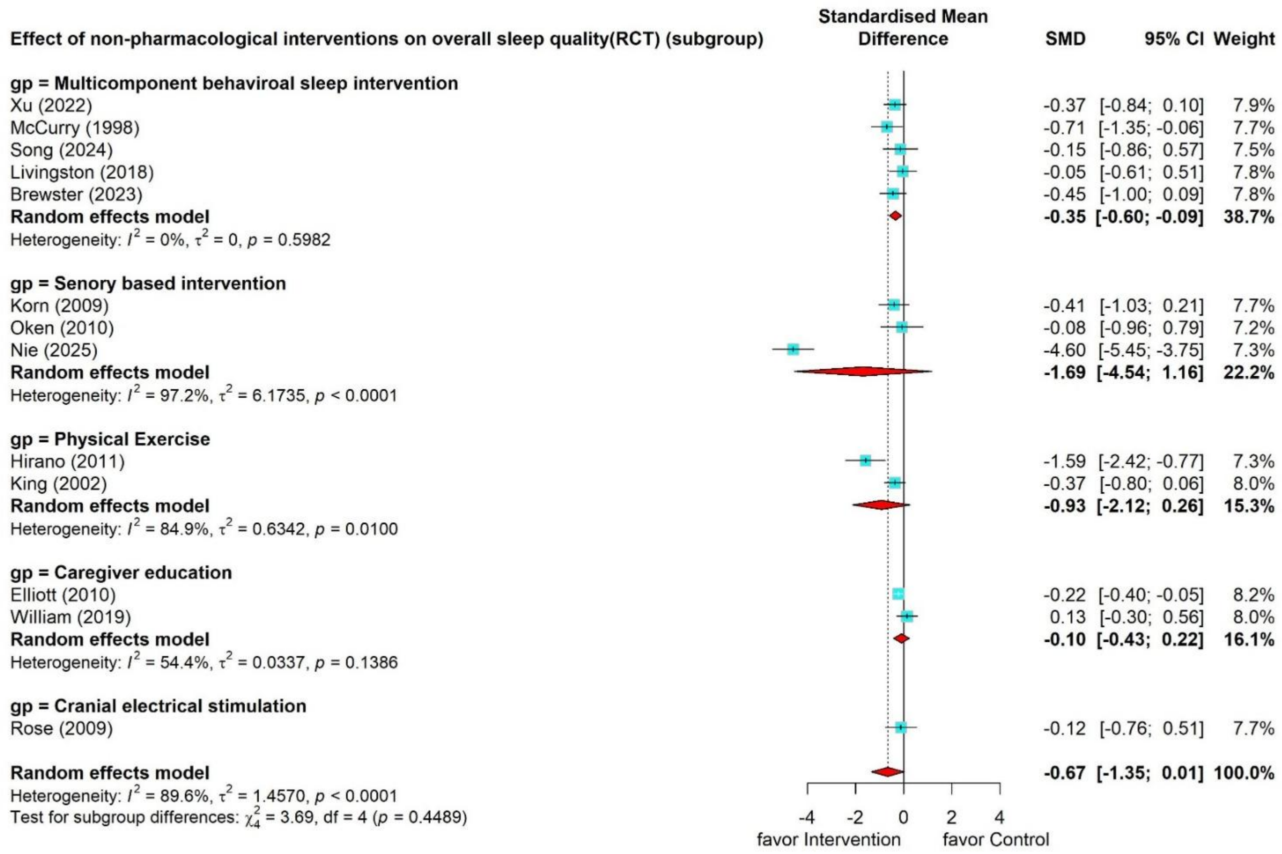

Supplementary File 7. Subgroup analysis comparing subjective or objective measures on sleep latency.

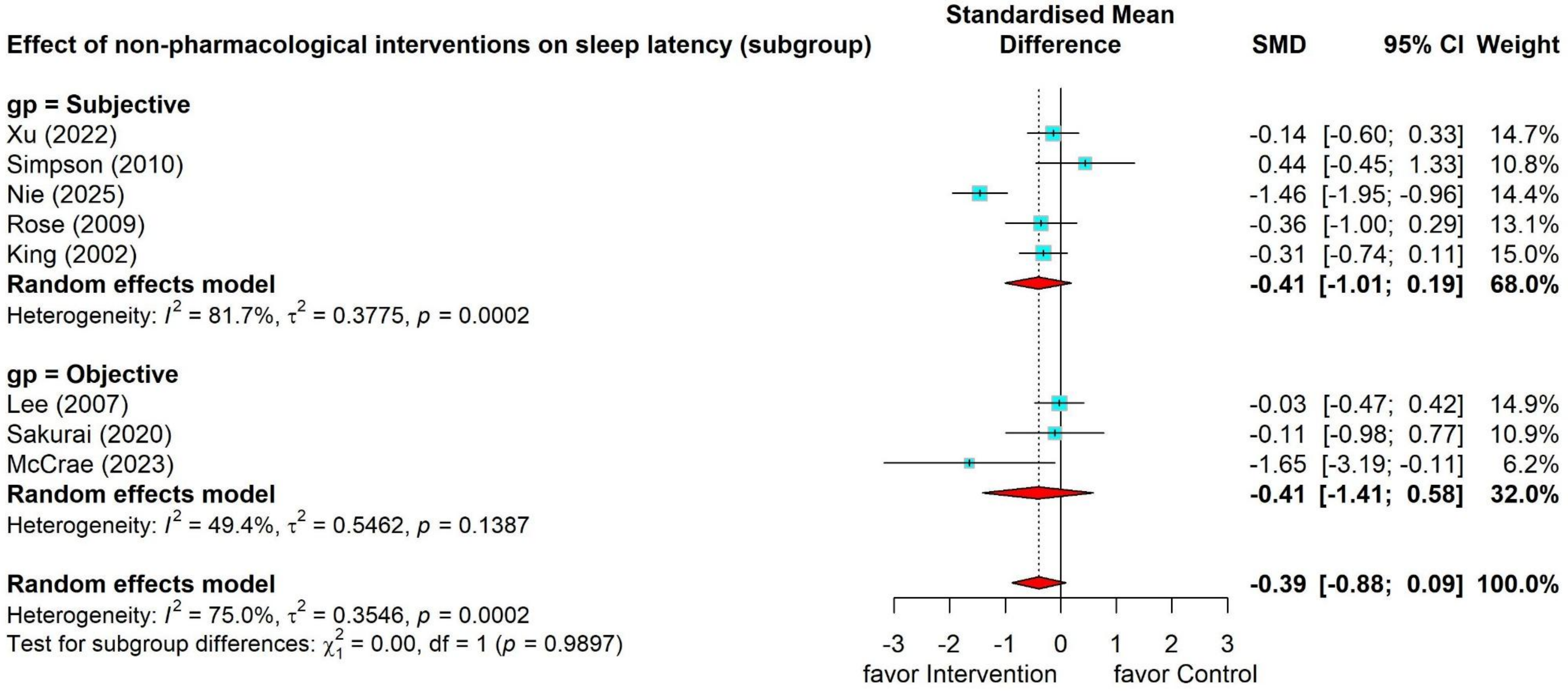

Supplementary File 8. Subgroup analysis comparing subjective or objective measures on wake after sleep onset.

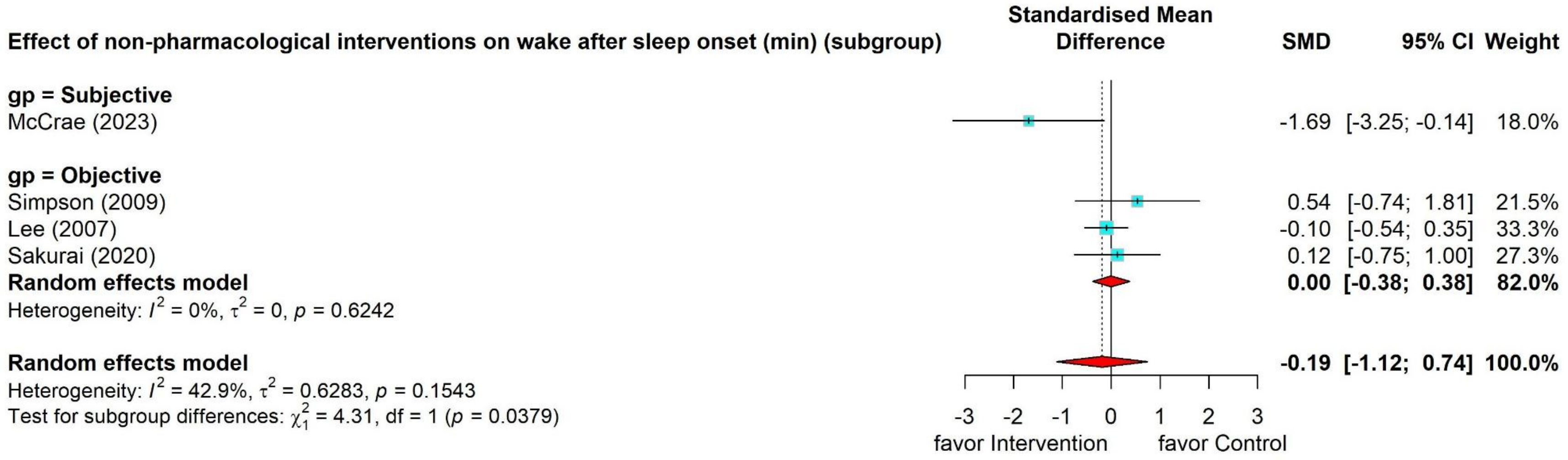

Supplementary File 9. Subgroup analysis comparing subjective or objective measures on sleep duration.

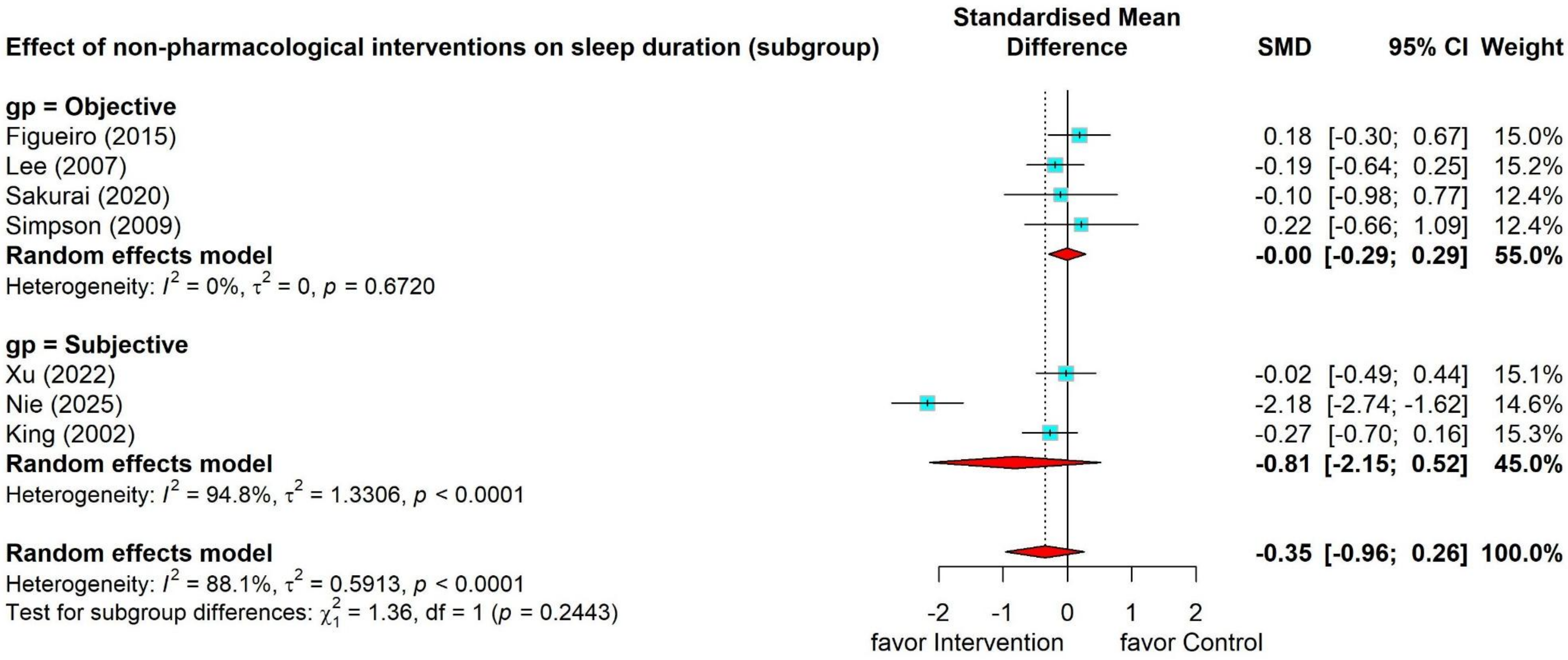

Supplement: gnag081_Supplementary_Data [file gnag081_supplementary_data.pdf]
